# Diagnostic accuracy of the partograph alert and action lines to predict adverse birth outcomes: a systematic review

**DOI:** 10.1111/1471-0528.15884

**Published:** 2019-08-18

**Authors:** M Bonet, OT Oladapo, JP Souza, AM Gülmezoglu

**Affiliations:** ^1^ UNDP/UNFPA/UNICEF/WHO/World Bank Special Programme of Research, Development and Research Training in Human Reproduction (HRP) Department of Reproductive Health and Research World Health Organization Geneva Switzerland; ^2^ Department of Social Medicine Ribeirão Preto Medical School University of São Paulo Ribeirão Preto Brazil

**Keywords:** Alert line, childbirth, diagnostic accuracy, partograph

## Abstract

**Background:**

There are questions about the use of the ‘one‐centimetre per hour rule’ as a valid benchmark for assessing the adequacy of labour progress.

**Objectives:**

To determine the accuracy of the alert (1‐cm/hour) and action lines of the cervicograph in the partograph to predict adverse birth outcomes among women in first stage of labour.

**Search strategy:**

PubMed, EMBASE, CINAHL, POPLINE, Global Health Library, and reference lists of eligible studies.

**Selection criteria:**

Observational studies and other study designs reporting data on the correlation between the alert line status of women in labour and the occurrence of adverse birth outcomes.

**Data collection and analysis:**

Two reviewers at a time independently identified eligible studies and independently abstracted data including population characteristics and maternal and perinatal outcomes.

**Main results:**

Thirteen studies in which 20 471 women participated were included in the review. The percentage of women crossing the alert line varied from 8 to 76% for all maternal or perinatal outcomes. No study showed a robust diagnostic test accuracy profile for any of the selected outcomes.

**Conclusions:**

This systematic review does not support the use of the cervical dilatation over time (at a threshold of 1 cm/h during active first stage) to identify women at risk of adverse birth outcomes.

**Tweetable abstract:**

Alert line of partograph does not identify women at risk of adverse birth outcomes.

## Tweetable abstract

Alert line of partograph does not identify women at risk of adverse birth outcomes.

## Introduction

Although most women and their babies are considered to be at low risk of complications at the onset of labour,^1^, ^2^ the time around childbirth is associated with the highest risk of maternal and perinatal mortality and morbidity.^3^, ^4^, ^5^ Unfortunately, the task of identifying pregnant women at risk of developing complications through labour, birth, and immediate postpartum is not trivial.^6^ Even in high‐resource settings, between 20 and 30% of low‐risk women present unexpected intrapartum complications such as dystocia, postpartum haemorrhage, infection, fetal distress or neonatal complications that would require specific obstetric or neonatal care.^1^, ^2^

For over two decades, the partograph has been the paper tool routinely applied to supporting decision‐making during labour with the aim of optimising time of interventions and referral.^7^ The central feature of the current partograph design is the cervicograph where cervical dilation is plotted, usually from 4 cm against time with an acceptable rate of dilatation at 1 cm per hour as designated by the alert line and, supposedly, representing the slowest tenth centile of nulliparous women in labour.^8^, ^9^ The action line was drawn at intervals of 4 hours after the alert line and was meant to identify abnormally slow labours and trigger review by medical staff with a view to augmentation, termination of labour or supportive therapy for women crossing this line.

Since the 1990s, WHO has promoted the use of the partograph during active phase of labour with a 4‐hour action line for monitoring progress of labour.^10^, ^11^, ^12^ However, more recently, several observational studies have raised questions about the use of the ‘one‐centimetre per hour rule’ as a valid benchmark for assessing the adequacy of labour progress.^13^, ^14^, ^15^

In this context, there is a need for a systematic assessment of the utility of the cervicograph alert and action lines in identifying women at higher risks of complications due to slow labour progression and need of interventions to reduce their risks of adverse birth outcomes. Therefore, we conducted a systematic review to determine the accuracy of the alert (1‐cm/h) and action lines of the cervicograph in the partograph to predict adverse birth outcomes among women in first stage of labour.

## Methods

This systematic review was conducted in accordance with the PRISMA guidelines,^16^ and followed a protocol, as described below.

### Eligibility criteria and search strategies

The review identified any study design where data showing the correlation between the alert line status of women in labour and the occurrence of adverse birth outcomes were reported, regardless of when the alert line was plotted (3 or 4 cm). Published or unpublished randomised controlled trials, diagnostic test accuracy studies, cross‐sectional studies, and longitudinal studies (retrospective or prospective) were considered eligible for inclusion if they used the WHO partograph, or any modified version of the WHO partograph, with alert and action lines at 1‐cm/h cervical dilatation rate threshold and defined a population of nulliparous and/or parous women with near‐term or term singleton pregnancy. Women considered at risk of developing complications during labour and childbirth, including women having presented complications during pregnancy, twin pregnancies or non‐cephalic presentations, were not excluded. No restriction based on sample size or number of participants with the outcome of interest was applied.

PubMed, EMBASE, CINAHL, POPLINE, Global Health Library, and reference lists of eligible studies were searched for potentially eligible studies. No restrictions related to publication status, date or language were applied. The literature search in electronic databases was carried out in April 2017. The search was updated in February 2019. The search strategy used a combination of the following terms, expanded and adapted for each database: ‘partograph’, ‘partogram’, ‘alert line’, and ‘birth outcomes’. Details of the search strategy are provided in Appendix S1. We contacted authors for ongoing and unpublished studies.

### Study selection, data collection, data items, and risk of bias

All citations identified through the electronic search were downloaded into reference management software, and duplicates were removed. All titles and abstracts were screened in duplicate by three independent reviewers (JPS, OTO, MB) considering the eligibility criteria. Full texts of potential eligible articles were assessed independently by two reviewers at a time (JPS, OTO, MB). Data were extracted using a standardised electronic table developed for this review and based on adapted criteria of the STARD (Standards for Reporting Diagnostic Accuracy Studies).^17^ Extracted data were double‐checked by a second reviewer (JPS or MB). Discrepancies on inclusions and/or data extraction were resolved through discussion or, if required, by the third reviewer.

Data extracted included the following domains: general information (author, title, publication date, country(ies) where the study took place, sample size); source of data; characteristics of participants (participant eligibility and recruitment method, participant characteristics, interventions during labour, study dates); description of use of partograph (including cervical dilatation to start plotting, time intervals between the alert and the action lines); adverse birth outcomes (definition of outcomes and measurement); and missing data. Study outcomes included fresh stillbirths, maternal (death, uterine rupture, organ dysfunction with dystocia), and neonatal outcomes (Apgar score at 1 and 5 minutes, resuscitation at birth, birth asphyxia/perinatal hypoxic‐ischaemic encephalopathy, labour ward deaths), as defined by authors. Data extracted from Diarra^18^ correspond to the full publication of the thesis^19^ as the journal publication has some data inconsistencies (confirmed with the authors).

For each study, the number of women in each of the following four categories was determined: women who crossed the alert line and had adverse birth outcomes, women who crossed the alert line and did not have adverse birth outcomes, women who did not cross the alert line and had adverse birth outcomes, women who did not cross the alert line and did not have adverse birth outcomes. Similarly, if available, the equivalent data for the action line were collected.

We developed a risk of bias assessment checklist, based on existing tools.^20^, ^21^ The assessment included the following domains: population selection (appropriate sampling and inclusions/exclusions), study attrition, measurement (temporality of the observations, outcomes measurement), and analysis (primary intent of the study). Quality of the studies was assessed by one reviewer (MB) and checked by a second reviewer (JPS). Discrepancies were resolved through discussion until consensus. The studies were assessed to be at low, high or unclear risk of bias based on whether the criterion is adequately fulfilled in the study or the study report does not provide sufficient information to allow for a clear judgement (Figure S1).

### Data analysis

The percentage of women crossing the alert line and the prevalence of adverse birth outcomes were determined for each study. The sensitivity, specificity, positive likelihood ratio, negative likelihood ratio, diagnostic odds ratio, and J statistic with their 95% confidence intervals were calculated to estimate the accuracy of the alert and action lines in the identification of women who would develop adverse maternal, fetal or neonatal outcomes. The diagnostic odds ratio is the ratio of the odds of disease in test positives relative to the odds of disease in test negatives: (TP × TN)/(FP × FN).^22^ The J statistic summarises the performance of a binary classifier^23^ and also expresses the proportion of ideal performance of a diagnostic test. It is calculated by (sensitivity + specificity) − 1, and a score close to ‘1’ indicates higher predictive capability. Diagnostic odds ratio was not computed for studies with zero values in one of the four categories described above. Interpretation of these statistics was performed as described in Table S2.

If outcome definitions were similar, the results of the studies were pooled by birth outcome, calculating summary sensitivity and specificity values. Results are also presented by outcome using paired forest plots. A composite outcome including fetal, maternal, and neonatal outcomes was also used if the data allowed differentiation of fresh stillbirths and at least one neonatal outcome. When multiple neonatal outcomes were reported, Apgar at 5 minutes or resuscitation was used to construct the composite outcome. A similar analysis considering the action line was carried out, and results are presented in Tables S4 and S5. The analyses were performed using an electronic spreadsheet (Microsoft Office Professional Plus 2010, Version 14.0) programmed with the standard formulas for diagnosis accuracy measures. Paired forest plots were designed using the scatter plots function in EXCEL 2010. Core outcome sets and patient involvement are not relevant for this review.

### Funding

The UNDP/UNFPA/UNICEF/WHO/World Bank Special Programme of Research, Development and Research Training in Human Reproduction (HRP), Department of Reproductive Health and Research, World Health Organization funded the preparation of this systematic review through a grant from the United States Agency for International Development (USAID), as part of the evidence base preparation towards the WHO recommendations on intrapartum care for a positive childbirth experience.

## Results

**Figure 1:**
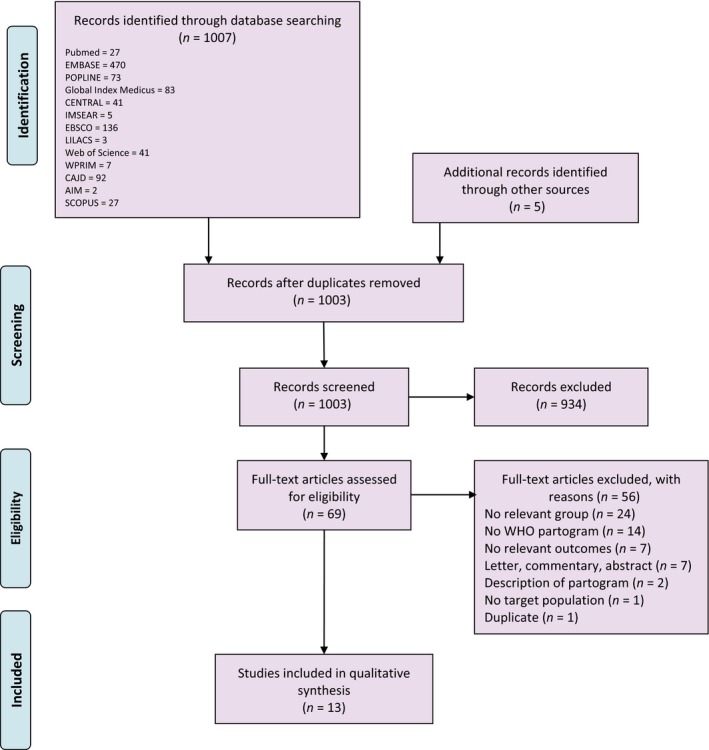
PRISMA flowchart.

The search strategies returned a total of 1007 potentially relevant citations (876 in April 2017 and additional 131 in February 2019), and 69 studies had full‐text manuscripts assessed for eligibility. A total of 13 studies in which 20 471 women participated were included in the review (Figure 1). The characteristics of included studies are provided in Table S1. Studies were conducted mainly in secondary or tertiary care facilities in Africa (12 106 women from Mali, Nigeria, Senegal, South Africa, and Uganda), the Americas (733 women from Brazil and Ecuador), Asia (7292 women from India, Indonesia, Malaysia, Thailand), and the Middle East (140 women from Iran). Two studies were conducted in the early 1990s and 10 on or after 2005. Three studies reported starting plotting cervical dilatation at 3 cm.^24^, ^25^, ^26^

Most of the studies included nulliparous (30–40% of the samples where specified) and parous women, one study included nulliparous women only,^27^ and the population was not specified in one study.^26^ Most women had no history of medical, surgical or obstetric problems and were included from 4 cm cervical dilatation, in spontaneous labours and vertex presentation.^18^, ^26^, ^28^, ^29^, ^30^, ^31^, ^32^ Two studies included spontaneous or induced labours and any fetal presentation.^25^, ^33^ One study^33^ also included in total 12% of women with pre‐labour complications during pregnancy. Women were attended by a range of providers, community health workers,^24^ midwives,^25^, ^28^, ^33^, ^34^ and obstetricians and midwives.^33^ In three studies, midwives were under supervision of an obstetrician^28^ or cases showing abnormal course of labour were re‐evaluated by senior obstetrician.^27^, ^35^ When reported, frequency of interventions during labour ranged from 7.6 to 46% for oxytocin augmentation, from 17% of artificial rupture of membranes to 100% in women in active phase of labour. Caesarean sections were performed in 2.7–60% of the births.

**Figure 2:**
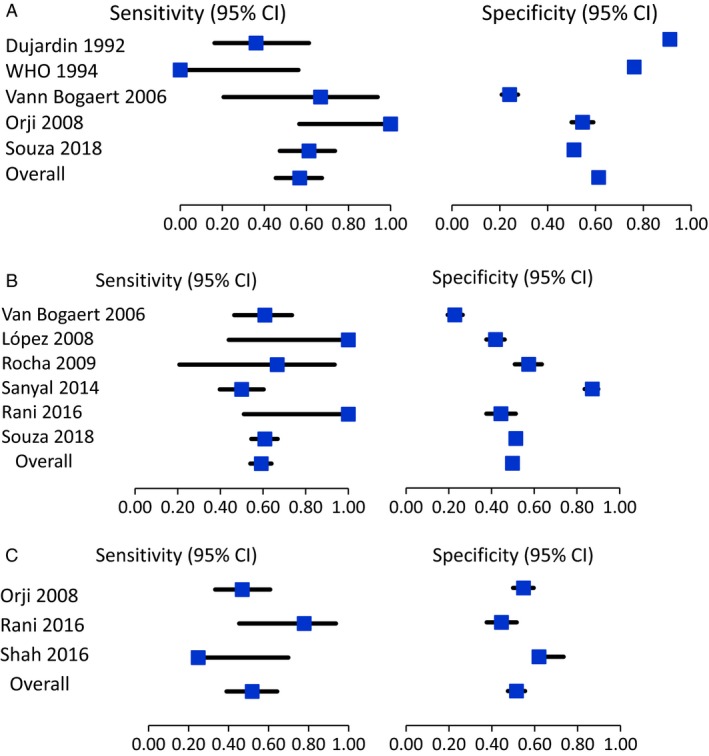
Sensitivity and specificity of the alert line for adverse fetal and neonatal outcomes. (A) Sensitivity and specificity of the alert line for adverse fetal (fresh stillbirths). (B) Sensitivity and specificity of the alert line for Apgar score <7 at 5 min. (C) Sensitivity and specificity of the alert line for birth asphyxia.

Table 1 and Figure 2A present the percentages of women who crossed the alert line, the prevalence of adverse fetal outcomes, and diagnosis accuracy measures of five included studies providing data on occurrence of fresh stillbirths (*n* = 17 029 women). The frequency of fresh stillbirths varied from 0 to 1.4% and alert line crossing varied from 9.3 to 75.9%. The sensitivity of the 1‐cm/h threshold (alert line) ranged from 36.0 to 100%, and the specificity from 24.1 to 91.1% for prediction of stillbirth during labour.

**Table 1:** Diagnostic test accuracy of the alert line for adverse fetal outcomes (fresh stillbirths)

Country (Year) references	Alert line status	Adverse fetal outcome		Percentage of alert line crossing	Prevalence of adverse fetal outcome	Sensitivity (95% CI)	Specificity (95% CI)	Positive likelihood ratio (95% CI)	Negative likelihood ratio (95% CI)	Diagnostic odds ratio (95% CI)	J statistic (95% CI)
		Present	Absent								
Senegal (1992)^24^	Crossed*	5	88	9.3	1.4%	36% (16.3–61.2)	91.1% (89.1–92.7)	4.0 (1.93–8.31)	0.71 (0.48–1.04)	5.67 (1.86–17.29)	26.8% (1.6– 52.0)
	Not crossed**	9	898								
Indonesia, Malaysia and Thailand (1994)^10^	Crossed	0	1532	23.8	0.0%	0 (0–56.2)	76.2% (75.2–77.2)	NA	1.31 (1.3–1.3)	NA	–23.78% (−24.8 to −22.7)
	Not crossed	3	4910								
South Africa (2006)^26^	Crossed	2	461	75.9	0.5%	66.7% (20.6–93.9)	24.1% (20.8–27.6)	0.88 (0.39–1.96)	1.39 (0.28– 6.91)	0.63 (0.06–7.04)	−9.3% (−62.7 to 44.2)
	Not crossed	1	146								
Nigeria (2008)^28^	Crossed	5	208	46.0	1.1%	100% (56.6–100)	54.6% (50.0–59.1)	2.2 (1.99–2.43)	NA	NA	54.6% (50.0– 59.1)
	Not crossed	0	250								
Nigeria and Uganda (2018)^32^	Crossed	30	4133	49.0	0.6%	61.2% (47.3–73.6)	51.0% (50.0–52.1)	1.25 (1.00–1.56)	0.76 (0.53–1.08)	1.65 (0.93–2.93)	12.3% (−1.4 to 24.9)
	Not crossed	19	4307								
**Overall**											
Fresh stillbirths	Crossed	42	6422	38.0	0.4%	56.8% (45.4–67.4)	62.1% (61.3–62.8)	1.50 (1.2–1.8)	0.70 (0.5–0.9)	2.15 (1.7–3.4)	18.8% (7.5– 30.1)
	Not crossed	32	10511								

NA, not applicable.

*Did not separate fetal death occurring before admission; outcome unknown for five women who crossed the line and two macerated stillbirths excluded from the denominator.

**Outcome unknown for three women who did not cross the line and 12 macerated stillbirths excluded from the denominator.

The same measures described above for neonatal outcomes are presented in Table 2 and Figures 2B,C, including Apgar score <7 at 5 minutes, birth asphyxia, and neonatal mortality following failed resuscitation after birth. There is wide variation in the frequency of adverse neonatal outcomes across the included studies (0.6–17.2%), with between 19.2 and 75.9% of women crossing the alert lines. Sensitivity and specificity ranges are very large, and results showed poor accuracy for all neonatal outcomes. No study showed a robust diagnostic test accuracy profile (i.e. positive likelihood ratio >10, negative likelihood ratio <0.20, diagnostic odds ratio >100, J statistic >50–80%). Table S3 presents results for Apgar score <7 at 1 minute and neonatal resuscitation. Heterogeneity of definitions precluded summary estimates for neonatal resuscitation.

**Table 2:** Diagnostic test accuracy of the alert line for adverse neonatal outcomes

Country (Year) references	Alert line status	Adverse neonatal outcome*		Percentage of alert line crossing	Prevalence of adverse neonatal outcome	Sensitivity (95% CI)	Specificity (95% CI)	Positive likelihood ratio (95% CI)	Negative likelihood ratio (95% CI)	Diagnostic odds ratio (95% CI)	J statistic (95% CI)
		Present	Absent								
**Apgar Score at 5 min** <**7**											
South Africa (2006)^26^	Crossed	28	433	75.9%	7.6%	60.9%	22.8%	0.79	1.72	0.46	−16.3% (−30.8 to −1.8)
	Not crossed	18	128			(46.5–73.6)	(19.5–26.5)	(0.6–1.0)	(1.2–2.5)	(0.3–0.9)	
Ecuador (2008)^32^	Crossed	3	289	58.4%	0.6%	100.0% (43.9–100)	41.9% (37.6–46.2)	1.72 (1.6–1.9)	NA	NA	
	Not crossed	0	208								
Brazil (2009)^34^	Crossed	2	98	42.9%	1.3%	66.7% (20.8–93.6)	57.4% (50.9–63.6)	1.56 (0.7–3.5)	0.58 (0.1–2.9)	2.69 (0.2–30.1)	24.1% (−29.6 to 77.8)
	Not crossed	1	132								
India(2014)^27^	Crossed	43	53	19.2%	17.2%	50.0% (39.7–60.3)	87.2% (83.6–90.0)	3.91 (2.8–5.4)	0.57 (0.5–0.7)	6.81 (4.1–11.4)	37.20% (26.2–48.2%)
	Not crossed	43	361								
India (2016)^31^	Crossed	4	109	56.5%	2.0%	100.0% (51.0–100)	44.4% (37.6–51.4)	1.80 (1.6–2.0)	NA	NA	44.4% (37.4–51.3)
	Not crossed	0	87								
Nigeria and Uganda (2018)^33^	Crossed	143	3990	49.0%	2.8%	60.9% (54.5–66.9)	51.4% (50.3–52.5)	1.25 (1.1–1.4)	0.76 (0.7–0.9)	1.64 (1.3–2.1)	12.2% (5.9–18.6)
	Not crossed	92	4215								
Overall	Crossed	223	4972	49.6%	3.6%	59.2% (54.1–64.0)	50.8% (49.8–51.8)	1.20 (1.1–1.3)	0.80 (0.7–0.9)	1.49 (1.2–1.8)	9.9% (4.9–15.0)
	Not crossed	154	5131								
**Birth asphyxia**											
Nigeria (2008)^28^	Crossed	22	186	45.4%	10.3%	46.8% (33.3–60.8)	54.7% (49.9–59.5)	1.03 (0.8–1.4)	0.97 (0.7–1.3)	1.06 (0.6–2.0)	1.6% (−13.5 to 16.6)
	Not crossed	25	225								
India (2016)^31^	Crossed	7	106	56.5%	4.5%	77.8% (45.3–93.7)	44.5% (37.6–51.6)	1.4 (0.97–2.0)	0.5 (0.2–1.7)	2.81 (0.6–13.9)	22.3% (−5.8 to 50.3%)
	Not crossed	2	85								
India (2016)^30^	Crossed	7	79	32.8%	5.7%	46.7% (24.8–69.9)	68.0% (62.0–73.5)	1.46 (0.8–2.6)	0.78 (0.5–1.3)	1.86 (0.7–5.3)	14.7% (−11.2 to 40.6)
	Not crossed	8	168								
Overall	Crossed	29	292	48.8%	8.5%	51.8% (39.0–64.3)	51.5% (47.5–55.5)	1.07 (0.8–1.4)	0.94 (0.7–1.2)	1.14 (0.7–2.0)	3.3% (−10.4 to 17.0)
	Not crossed	27	310								
**Early neonatal mortality**											
South Africa (2006)^26^, **	Crossed	8	453	75.9%	3.0%	44.4% (24.6–66.3)	23.1% (19.9–26.7)	0.58 (0.3–0.97)	2.41 (1.6–3.7)	0.24 (0.1–0.6)	−32.5% (–55.7 to −9.3)
	Not crossed	10	136								

NA, not applicable.

*Stillbirths and unknown outcomes excluded from the denominator as follows. Dujardin^24^: outcome unknown for 33 neonates and 28 stillbirths excluded; WHO^12^: three stillbirths excluded; Van Bogaert^26^: three stillbirths excluded Orji^28^: five stillbirths excluded; Souza^33^: 49 stillbirths excluded).

**Deaths resulted from low Apgar scores and unsuccessful resuscitation.

The diagnostic test accuracy measures for the action line are presented in Tables S4 and S5, respectively, for fresh stillbirths and neonatal outcomes, with similar results. Results of the composite outcome including fetal, maternal, and neonatal outcomes are presented in Table S6.

## Discussion

### Main findings

In general, no study showed a robust diagnostic test accuracy profile of the alert and action lines for any of the outcomes studied.

### Strengths and limitations

To our knowledge, this is the first review assessing diagnostic accuracy of the partograph alert and action lines for the identification of women at risk of birth complications. Included studies covered populations with diverse obstetric history and characteristics, exposed to a range of healthcare practices and contexts. Most of included studies provided recent data from the last decade. These characteristics favour generalisability of our results to the current management of women in labour in low‐ and middle‐income countries. No eligible studies were conducted in high‐income countries. Large variations in prevalence of adverse outcomes and diagnostic test performance should be interpreted with caution. The interaction between prevalence and sensitivity and specificity should not be overlooked, particularly when the prevalence of the condition is low, and considering that the occurrence of false positives can erode the test performance.

Overall quality of the studies was low, mainly in relation to the inherent limitations in the design and conduct of the primary studies. Selection of populations and definitions of outcomes included in the review varied, in particular for resuscitation and low Apgar. We hypothesised that when timing of resuscitation was not reported, resuscitation referred to the period immediately after birth, but cases reported could have referred to any moment during hospital stay after birth and may not be directly related to childbirth. In addition, the review focused on the accuracy of the partograph line to identify women and fetuses at risk of birth complications and did not include the same assessment for other variables registered on the partograph. We were unable to assess the usefulness of the alert line in optimising referral of women in labour from rural or primary healthcare facilities to secondary or tertiary units, as only one of the studies identified was conducted in a peripheral hospital.^24^ Finally, we did not assess new labour curves and consequent new partographs (i.e. those not abiding to the 1 cm/h dilatation rate rule).^13^, ^14^, ^36^

### Interpretation

The largest study included in this review found a mild increase in the risk of adverse birth outcomes in slow labours compared with fast labour.^33^ The authors recognised that fetal and early neonatal outcomes are much more likely to be impacted by events that are not related to cervical dilatation rate. Those authors found that although other partograph variables were associated with mild to moderately increased odds of severe adverse birth outcomes, these also had poor diagnostic performance for the prediction of severe birth outcomes.

In light of the findings of another systematic review on cervical dilatation patterns,^37^ it is not surprising that the alert and action line failed to identify women at higher risk of adverse outcomes. That review showed that it is not uncommon for women to experience long labours and still have good birth outcomes. It also showed that labour progression in women with normal birth outcomes is not linear, and that dilatation rates before 5 or 6 cm may be slower than 1 cm/h, but cervical dilatation rates may be faster after 5–6 cm. These findings may help to explain our results: if it is common for women with good birth outcomes to have labours slower than 1 cm/h, they could have crossed the partograph alert line between 3 and 4 cm and up to 6 cm.

Thus, overall, studies included in this review showed high proportions of women crossing the alert line, in contrast to the expected rate of alert line crossing which was supposed to represent the slowest 10% of labour progress in primigravidas.^9^ In this sense, the use of the alert line alone to trigger referrals from peripheral to higher level hospitals may have unnecessarily increased referrals of women who otherwise had labours that were progressing normally. This may have had huge emotional, physical, and costs implications not only for the women, the fetus, and their families, but also for healthcare providers in referring and receiving facilities and the health system, in particular in places where referral systems are suboptimal or where higher level maternity units are overcrowded or understaffed.^38^

Included studies did not systematically report whether protocols were in place for assessment of labour progress, fetal vital status, and birth asphyxia, which could have affected the management of complications and the reported adverse birth outcomes across studies. Differences in protocols used along with the partograph to manage labour, including labour dystocia as depicted by the partograph lines, may have affected progress of labour and outcomes, and increased the risks of iatrogenic adverse outcomes, related to the use of oxytocin, caesarean section or suboptimal referrals, or decreased that risk if interventions to accelerate labour had a positive effect in averting adverse outcomes. The need for intensified monitoring and specialised care, e.g. to monitor an augmented labour or to perform a caesarean section, may have further increased costs and contributed to staff fatigue and burn out in health facilities.

This review does not question the importance of labour monitoring for all women and fetuses. Healthcare professionals should continue to plot other partograph parameters to monitor the well‐being of the woman and her baby, and identify risks for adverse birth outcomes until new tools are proven to be more or equally effective. The Cochrane review^7^ on the partograph recognises that its use may provide some benefits in terms of quality of care benefits. The alert line continues to be relevant for care of women in healthcare facilities where interventions such as augmentation and caesarean section cannot be performed and where referral‐level facilities are difficult to reach.

Recently, some organisations have revised their labour definitions of active first stage of labour, to start at 5 or 6 cm,^1^, ^39^, ^40^ and to accommodate a cervical dilatation rate slower than 1 cm/h as the normal threshold. New tools have been developed to allow for longer labours without intervention. However, assessment of these new definitions and tools is limited and have included small samples,^41^, ^42^ conducted in high‐income countries or focusing on reduction of caesarean section.^36^, ^41^, ^42^, ^43^ Others have focused on evaluation of the impact of new labour definitions on labour outcomes interventions. Two observational studies conducted in the USA yielded to different findings in terms of frequency of maternal and neonatal morbidity, and reduction of caesarean sections.^42^, ^44^ There is a need to assess the added value of different designs of labour monitoring tools in the improvement of birth outcomes and reduction of unnecessary interventions during labour. This should also include cost‐benefit analysis, considering the potential reduction in unnecessary interventions.^44^

## Conclusion

The body of evidence compiled in this systematic review does not support the use of a threshold of 1 cm/h of cervical dilatation to identify women at risk of adverse birth outcomes. Women with fast labours (i.e. not crossing the alert, or action, line) are not free of risk of adverse birth outcomes. There is a need to identify optimal benchmarks for assessing progress of labour to guide birth attendants on when best to intervene to reduce adverse birth outcomes for women and infants.

### Disclosure of interests

OTO, JPS, and AMG participated in a large study on labour monitoring and action with a component that included assessment of diagnostic accuracy of labour curves in the partograph. MB has no conflicts of interest to declare. Completed disclosure of interests forms are available to view online as supporting information.

### Contribution to authorship

OTO and JPS conceived the review and drafted the protocol of the review, with input from MB and AMG. OTO worked with the WHO information specialists to build the search strategies and undertake the searches. OTO, JPS, and MBS performed the initial screening of search outputs, identified eligible studies, and extracted data. JPS and MB performed the data analysis with inputs from the other authors, and MBS wrote the first draft of the paper. All authors contributed to revising the final version and approved the manuscript for publication.

### Funding

The UNDP/UNFPA/UNICEF/WHO/World Bank Special Programme of Research, Development and Research Training in Human Reproduction (HRP), Department of Reproductive Health and Research, World Health Organization funded the preparation of this systematic review through a grant from the United States Agency for International Development (USAID), as part of the evidence base preparation towards the WHO recommendations on intrapartum care for a positive childbirth experience.

## Supporting information


**Figure S1**. Risk of bias assessment.

**Table S1**. Description of included studies.

**Table S2**. Suggested interpretation of diagnostic accuracy statistics.

**Table S3**. Diagnostic test accuracy of the alert line for adverse neonatal outcomes.

**Table S4**. Diagnostic test accuracy of the action line for adverse fetal outcomes.

**Table S5**. Diagnostic test accuracy of the action line for adverse neonatal outcomes.

**Table S6**. Diagnostic test accuracy of the alert line for composite adverse birth outcomes.

**Appendix S1**. Search strategy.

## References

[1] National Institute for Health and Clinical Excellence . Intrapartum care for healthy women and babies. 2014 [https://www.nice.org.uk/guidance/cg190]. Accessed 12 February 2019.

[2] Danilack V , Nunes A , Phipps M . Unexpected complications of low‐risk pregnancies in the United States. Am J Obstet Gynecol 2015;212:809.e1–6. 26042957 10.1016/j.ajog.2015.03.038 PMC4728153

[3] Lawn JE , Kinney M , Lee AC , Chopra M , Donnay F , Paul VK , et al. Reducing intrapartum‐related deaths and disability: can the health system deliver? Int J Gynaecol Obstet 2009;107(Suppl. 1):S123–40, s40‐2. 19815205 10.1016/j.ijgo.2009.07.021

[4] Lee AC , Kozuki N , Blencowe H , Vos T , Bahalim A , Darmstadt GL , et al. Intrapartum‐related neonatal encephalopathy incidence and impairment at regional and global levels for 2010 with trends from 1990. Pediatr Res 2013;74(Suppl 1):50–72. 24366463 10.1038/pr.2013.206 PMC3873711

[5] Bhutta ZA , Das JK , Bahl R , Lawn JE , Salam RA , Paul VK , et al. Can available interventions end preventable deaths in mothers, newborn babies, and stillbirths, and at what cost? Lancet 2014;384:347–70. 24853604 10.1016/S0140-6736(14)60792-3

[6] Wall SN , Lee AC , Carlo W , Goldenberg R , Niermeyer S , Darmstadt GL , et al. Reducing intrapartum‐related neonatal deaths in low‐ and middle‐income countries—what works? Semin Perinatol 2010;34:395–407. 21094414 10.1053/j.semperi.2010.09.009

[7] Lavender T , Hart A , Smyth RM . Effect of partogram use on outcomes for women in spontaneous labour at term. Cochrane Database Syst Rev 2013;(7):CD005461. 23843091 10.1002/14651858.CD005461.pub4

[8] Friedman E . The graphic analysis of labor. Am J Obstet Gynecol 1954;68:1568–75. 13207246 10.1016/0002-9378(54)90311-7

[9] Philpott RH , Castle WM . Cervicographs in the management of labour in primigravidae. I. The alert line for detecting abnormal labour. J Obstet Gynaecol Br Commonwealth 1972;79:592–8. 10.1111/j.1471-0528.1972.tb14207.x 5043422

[10] World Health Organization . World Health Organization partograph in management of labour. World Health Organization maternal health and safe motherhood programme. Lancet 1994;343:1399–404. 7910888

[11] World Health Organization . Pregnancy, Childbirth, Postpartum and Newborn Care: A Guide for Essential Practice, 3rd edn Geneva: WHO; 2015. 26561684

[12] World Health Organization . WHO recommendations for augmentation of labour; 2014 [https://www.who.int/reproductivehealth/publications/maternal_perinatal_health/augmentation-labour/en/]. Accessed 12 February 2019. 25506951

[13] Zhang J , Landy HJ , Ware Branch D , Burkman R , Haberman S , Gregory KD , et al. Contemporary patterns of spontaneous labor with normal neonatal outcomes. Obstet Gynecol 2010;116:1281–7. 21099592 10.1097/AOG.0b013e3181fdef6e PMC3660040

[14] Neal JL , Lowe NK . Physiologic partograph to improve birth safety and outcomes among low‐risk, nulliparous women with spontaneous labor onset. Med Hypotheses 2012;78:319–26. 22138426 10.1016/j.mehy.2011.11.012 PMC3254242

[15] Hamilton EF , Warrick PA , Collins K , Smith S , Garite TJ . Assessing first‐stage labor progression and its relationship to complications. Am J Obstet Gynecol 2016;214:358.e1‐8. 26478103 10.1016/j.ajog.2015.10.016

[16] Moher D , Liberati A , Tetzlaff J , Altman DG . Preferred reporting items for systematic reviews and meta‐analyses: the PRISMA statement. PLoS Med 2009;6:e1000097. 19621072 10.1371/journal.pmed.1000097 PMC2707599

[17] Cohen JF , Korevaar DA , Altman DG , Bruns DE , Gatsonis CA , Hooft L , et al. STARD 2015 guidelines for reporting diagnostic accuracy studies: explanation and elaboration. BMJ Open 2016;6:e012799. 10.1136/bmjopen-2016-012799 PMC5128957 28137831

[18] Diarra I , Camara S , Maiga MK . Evaluation de l'utilisation du partogramme à la maternité du centre de santé de référence de la commune V du district de Bamako. Mali Med 2009;24:10–3. 19666359

[19] Camara MS . Evaluation de l`utilisation du partogramme au Centre de Santé de Référence de la Commune V du District de Bamako: Faculté de Médecine, de Pharmacie et d'Odonto‐Stomatologie, Universite de Bamako, Mali; 2007. [http://www.keneya.net/fmpos/theses/2007/med/pdf/07M235.pdf]. Accessed 12 February 2019.

[20] Whiting PF , Rutjes AW , Westwood ME , Mallett S , Deeks JJ , Reitsma JB , et al. QUADAS‐2: a revised tool for the quality assessment of diagnostic accuracy studies. Ann Intern Med 2011;155:529–36. 22007046 10.7326/0003-4819-155-8-201110180-00009

[21] Hayden JA , van der Windt DA , Cartwright JL , Cote P , Bombardier C . Assessing bias in studies of prognostic factors. Ann Intern Med 2013;158:280–6. 23420236 10.7326/0003-4819-158-4-201302190-00009

[22] Glas AS , Lijmer JG , Prins MH , Bonsel GJ , Bossuyt PM . The diagnostic odds ratio: a single indicator of test performance. J Clin Epidemiol 2003;56:1129–35. 14615004 10.1016/s0895-4356(03)00177-x

[23] Youden WJ . Index for rating diagnostic tests. Cancer 1950;3:32–5. 15405679 10.1002/1097-0142(1950)3:1<32::aid-cncr2820030106>3.0.co;2-3

[24] Dujardin B , De Schampheleire I , Sene H , Ndiaye F . Value of the alert and action lines on the partogram. Lancet 1992;339(8805):1336–8. 1350000 10.1016/0140-6736(92)91969-f

[25] World Health Organization . Maternal Health and Safe Motherhood Programme. The Partograph: the application of the WHO partograph in the management of labour, report of a WHO multicentre study, 1990‐1991. 1994.

[26] Van Bogaert LJ . The partogram's result and neonatal outcome. J Obstet Gynaecol 2006;26:321–4. 16753681 10.1080/01443610600594963

[27] Uugc S , Goswami S , Mukhopadhyay P . The role of partograph in the outcome of spontaneous labor. NJOG 2014;17:52–7.

[28] Orji E . Evaluating progress of labor in nulliparas and multiparas using the modified WHO partograph. Int J Gynecol Obstet 2008;102:249–52. 10.1016/j.ijgo.2008.04.024 18603248

[29] Bolbol HN , Ebrahimi H , Delvarian ZM , Hassani MR . Evaluation of WHO's partogram alert line for prediction of the APGAR score at the first minute after birth. J Shahrekord Univ Med Sci 2006;8:50–7, 8.

[30] Shah N , Maitra N , Pagi SL . Evaluating role of parity in progress of labour and its outcome using modified WHO partograph. Int J Reprod Contracept Obstet Gynecol 2016;5:860–3.

[31] Uvgc R , Laxmi BV . Effect of partographic monitoring on outcomes for women in spontaneous labour at term. IAIM 2016;3:314–20.

[32] López CAF . Estudio comparativo entre el partograma del clap y el partograma de la oms en embarazadas del hospital Vicente Corral Moscoso de Cuenca, Ecuador. 2008:60.

[33] Souza JP , Oladapo OT , Fawole B , Mugerwa K , Reis R , Barbosa‐Junior F , et al. Cervical dilatation over time is a poor predictor of severe adverse birth outcomes: a diagnostic accuracy study. BJOG 2018;125:991–1000. 29498187 10.1111/1471-0528.15205 PMC6032950

[34] Rocha IM , de Oliveira SM , Schneck CA , Riesco ML , da Costa AS . The partogram as an instrument to analyze care during labor and delivery. IJBAR 2009;43: 880–8. 10.1590/s0080-62342009000400020 20085159

[35] Shinde KK , Bangal VB , Singh RK . Study of course of labour by modified WHO partograph. IJBAR 2012;3:291–6.

[36] Bernitz S , Dalbye R , Oian P , Zhang J , Eggebo TM , Blix E . Study protocol: the Labor Progression Study, LAPS ‐ does the use of a dynamic progression guideline in labor reduce the rate of intrapartum cesarean sections in nulliparous women? A multicenter, cluster randomized trial in Norway. BMC Pregnancy Childbirth 2017;17:370. 29132336 10.1186/s12884-017-1553-8 PMC5683365

[37] Oladapo OT , Diaz V , Bonet M , Abalos E , Thwin SS , Souza H , et al. Cervical dilatation patterns of ‘low‐risk’ women with spontaneous labour and normal perinatal outcomes: a systematic review. BJOG 2018;125:944–54. 28892266 10.1111/1471-0528.14930 PMC6033146

[38] Elmusharaf K , Byrne E , AbuAgla A , AbdelRahim A , Manandhar M , Sondorp E , et al. Patterns and determinants of pathways to reach comprehensive emergency obstetric and neonatal care (CEmONC) in South Sudan: qualitative diagrammatic pathway analysis. BMC Pregnancy Childbirth 2017;17:278. 28851308 10.1186/s12884-017-1463-9 PMC5576292

[39] Haute Autorité de Santé . Recommandation de bonne pratique. Accouchement normal :accompagnement de la physiologie et interventions médicales. Méthode Recommandations pour la pratique clinique. France: HAS; 2017 [https://www.has-sante.fr/portail/upload/docs/application/pdf/2018-01/accouchement_normal_-_recommandations.pdf]. Accessed 12 February 2019.

[40] Caughey AB , Cahill AG , Guise JM , Rouse DJ . Safe prevention of the primary cesarean delivery. Am J Obstet Gynecol 2014;210:179–93. 24565430 10.1016/j.ajog.2014.01.026

[41] Neal JL , Lowe NK , Nacht AS , Koschoreck K , Anderson J . Pilot study of physiologic partograph use among low‐risk, nulliparous women with spontaneous labor onset. J Midwifery Women's Health 2016;61:235–41. 26917257 10.1111/jmwh.12442

[42] Wilson‐Leedy JG , DiSilvestro AJ , Repke JT , Pauli JM . Reduction in the cesarean delivery rate after obstetric care consensus guideline implementation. Obstet Gynecol 2016;128:145–52. 27275806 10.1097/AOG.0000000000001488

[43] Neal JL , Lowe NK , Phillippi JC , Ryan SL , Knupp AM , Dietrich MS , et al. Likelihood of cesarean delivery after applying leading active labor diagnostic guidelines. Birth 2017;44:128–36. 28198038 10.1111/birt.12274 PMC7608623

[44] Rosenbloom JI , Stout MJ , Tuuli MG , Woolfolk CL , Lopez JD , Macones GA , et al. New labor management guidelines and changes in cesarean delivery patterns. Am J Obstet Gynecol 2017;217:689.e1–.e8. 29037483 10.1016/j.ajog.2017.10.007 PMC5712240

