# Supplementary material for: Diagnostic accuracy of the partograph alert and action lines to predict adverse birth outcomes: a systematic review
Source: BJOG. 2019 Aug 18;126(13):1524–33. doi: 10.1111/1471-0528.15884 (PMC6899985; doi:10.1111/1471-0528.15884)
Supplement: Supplementary file 1 — Figure S1. Risk of bias assessment. [file BJO-126-1524-s001.pdf]

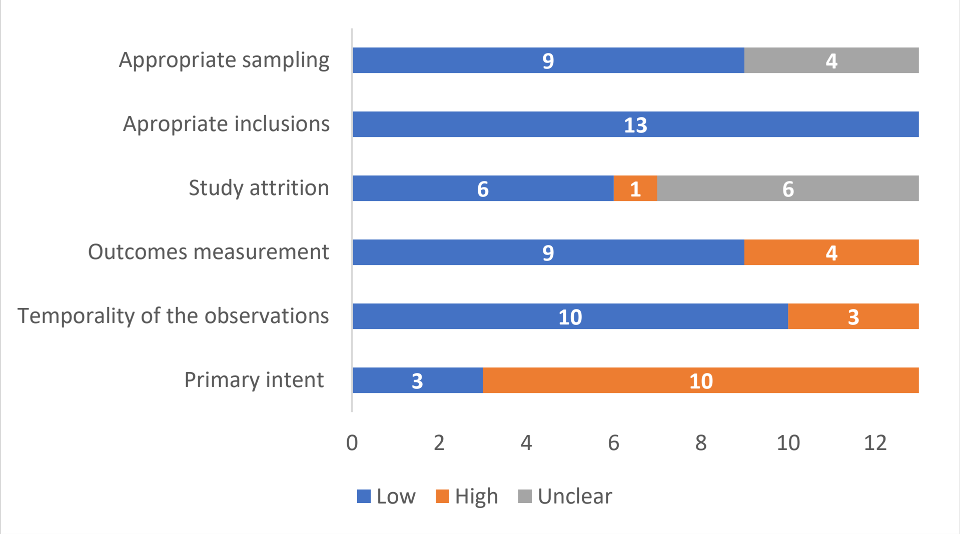

Figure S1. Risk of bias assessment: number of studies with low, high or unclear risk of bias.

Definitions used: Appropriate sampling: consecutive or random sample, no case-control; Appropriate inclusions and exclusions: sample represents the population of interest; Study attrition: proportion of study sample completing the study and providing outcome data is adequate, no important differences between participants and non-participants; Outcomes measurement: Adequate description/definition of all outcomes of interest; Temporality of the observations: direct observations vs retrospective review of medical chart; Primary intent of the research question: evaluation of diagnostic accuracy using at least one measure of accuracy.
