# Supplementary material for: Diagnostic accuracy of the partograph alert and action lines to predict adverse birth outcomes: a systematic review
Source: BJOG. 2019 Aug 18;126(13):1524–33. doi: 10.1111/1471-0528.15884 (PMC6899985; doi:10.1111/1471-0528.15884)
Supplement: Supplementary file 2 — Table S1. Description of included studies. [file BJO-126-1524-s002.pdf]

Table S1. Characteristics of included studies and study populations

Country (Author, year)	Sample size	Study design	Data collection	Setting	Staff assessing labour progress	Characteristics of women included	Exclusions
Senegal (Dujardin, 1992)	1022	Cross-sectional	Prospective 1990	4 peripheral maternity clinics	midwives and CHW	All pregnant women presenting to participating facilities.	Partograms were unavailable for 551 (35%) women because of unexpected delivery at home or en route (145), emergency transfer upon admission (12), or expulsion phase or complete dilatation upon admission (394). For the remaining 15 cases, partograms either were not completed or had been lost.
Indonesia, Malaysia and Thailand (WHO, 1994)	6445	Cluster trial	1990-1991	4 pairs of hospitals district general hospitals, urban setting	midwifes	Nulliparous (37%) and parous women admitted in active phase, spontaneous or induced labours. Normal group of women defined as those for whom labour complications could not be anticipated	Preterm less than 34 weeks, cervix dilated 9 or 10 cm, elective caesarean sections, emergency caesarean section within one hour of admission
Iran (Bolbol-Haghighi, 2006)	140	Cross-sectional	Prospective 2011	One maternity hospital	midwifes	Nulliparous and parous women, uncomplicated pregnancy, spontaneous labour, maximal dilatation of 3 cm upon admission	
South Africa (Van Bogaert, 2006)	610	Cross-sectional	Retrospective no year provided	One district hospital	not available	Spontaneous vaginal or instrumental births and emergency caesarean. Parity not specified	
Ecuador (López, 2008)	500	Cross-sectional	Prospective 2004-2005	One hospital	not available	Nulliparous and parous women, spontaneous labour, admitted between 4 and 9 cm cervical dilatation	Emergency caesarean section, preterm, stillbirths
Nigeria (Orji, 2008)	463	Cross-sectional	Prospective 2007	One University teaching hospital	midwives under supervision of an obstetrician	Nulliparous and parous women, uncomplicated, spontaneous labour with cervical dilatation at 4 cm or more.	Premature labor, eclampsia, antepartum hemorrhage, induced labor, or multiple pregnancies
Mali (Diarra 2009)	1522	Cross-sectional	Retrospective 2005	One referral hospital	not available	Nulliparous (32%) and parous women, admitted in labour before 8 cm cervical dilatation	Planned caesarean section, cervical dilatation 8cm or more
Brazil (Rocha 2009)	233	Cross-sectional	Retrospective 2004-2005	One maternity hospital (Normal Childbirth Center)	midwifes	Nulliparous (40%) and parous women, spontaneous labour, childbirth occurred at least two hours after admission, alert and action lines marked on the partogram; partogram marked correctly according to the model proposed by the Health Ministry	

India (Shinde, 2012)	100	Cross-sectional, randomly selected cases	Prospective 2009-2011	One eaching tertiary hospital		Nulliparous and parous women, presenting to labour room	Women with anemia, hypertension, diabetes and immune compromised status, preterm labour, previous LSCS, postdatism, multiple pregnancies, antepartum hemorrhage (APH), intrauterine growth restriction, (IUGR), premature rupture of membranes, (PROM), intrauterine fetal death (IUFD)
India (Sanyal, 2014)	500	Cross-sectional	Prospective 2011-2012	One teaching hospital	Cases showing abnormal course of labour were reevaluated by senior obstetrician	Nulliparous, uncomplicated pregnancy	Non vertex presenting part, known major fetal structural anomalies, previous uterine surgery, antepartum hemorrhage, premature rupture of membranes, intrauterine growth retardation, intra uterine fetal death, anemia, hypertension, diabetes, immunocompromised state
India (Rani, 2016)	200	Cross-sectional	Prospective 2011-2013	One maternity hospital	not available	Nulliparous and parous women, uncomplicated, spontaneous labour	PPH, oligohydramnios, anemia, heart disease, diabetes, teenage pregnancy, hypertensive disorder of pregnancy, renal disease, macrosomia, polyhydramnois, IUGR, placenta previa for vaginal delivery (type I and type II anterior), liver disease, malpresentation breech, multiple pregnancy, preterm labour, scarred uterus, accidental haemorrhage, HIV /HBsAg
India (Shah, 2016)	247	Cross-sectional	Prospective 2012-2013	One general hospital	not available	Nulliparous and parous women, uncomplicated pregnancy, spontaneous labour, from 4cm dilatation	History of medical, surgical, or obstetric problems, history of taking analgesic drugs during pregnancy, and congenital fetal problems
Nigeria and Uganda (Souza, 2018)*	8489	Prospective Cohort	Prospective 2014-2015	13 hospitals with a minimum of 1,000 deliveries per year	Midwives, obstetricians or obstetric residents	Nulliparous (41%) and parous women, with spontaneous onset of labour presenting at cervical dilatation of ≤6 cm and those undergoing induction of labour	

*Characteristics of the population presented for the total sample of 9995 women

Table S1. Continued

Country (Author, year)	Fetal characteristics	Caesarean section %	Artificial rupture of membranes %	Oxytocin augmentation %	Other labour characteristics	Protocols in place	Start point for plotting cervicograph/ lag time of action line	Comments
Senegal (Dujardin, 1992)	NA	Not applicable	–	–	–		3cm/3h	Do not identify intra-uterine deaths at admission
Indonesia, Malaysia and Thailand (WHO, 1994)	Singleton and multiple pregnancies, over 34 weeks of gestation, all fetal presentations (89% spontaneous vertex vaginal birth)	2.7%	All women in active phase	7.6%	–	All facilities practising active management of labour. Protocol included rupture of membranes in active phase of labour.	3cm/4h	Includes augmentation with oxytocin, spontaneous or operative vaginal birth and caesarean sections. Reported also relevant outcomes on women admitted in latent phase. Separates intra-uterine deaths at admission and those occurring after admission
Iran (Bolbol-Haghighi, 2006)	Live fetus, singleton, at term, cephalic presentation 39.7 ± 6.92 weeks	–	–	15%	–		3cm/4h	–
South Africa (Van Bogaert, 2006)	NA	60% CS, 223 for prolonged labour, 90 fetal distress	–	–	–		3cm/4h	Resuscitation determined by an Apgar score at 1 min of less than 7.<br>Reported also 18 early neonatal deaths (10 left to the alert line, 8 right to the alert line)<br>The spontaneous vaginal deliveries were selected to match the distribution of the emergency CS in terms of speed of cervical dilatation on the partogram
Ecuador (López, 2008)	Live fetus, term (38-41 weeks), cephalic presentation Mean GA 39,4 ± 1,03	–	–	–	–		4cm/4h	–
Nigeria (Orji, 2008)	Singleton pregnancy, at term Mean birth weight:3250 grams, Mean GA: 39	24%	–	36%	–		4cm/4h	Includes augmentation with oxytocin, spontaneous or operative vaginal birth and caesarean sections. The 5 fresh stillbirths occurred in nulliparas whose labor was augmented because of a delay in labor progress.Tight coiling of the umbilical cord around the neck caused the fetal death in each case.
Mali (Diarra 2009)	88.5% with fundal height between 32-36 cm, 90% cephalic presentation	35%	–	–	–		4cm/4h	Additional information in Camara 2007 (http://www.keneya.net/fmpos/theses/2007/med/pdf/07M235.pdf): five maternal deaths and 6 uterine rupture but no information by group. Apgar score reported is at first minute. Reported 57 neonatal deaths and 5 maternal deaths.
Brazil (Rocha 2009)	Live fetus, singleton, at term, cephalic presentation	7%	53%	46%	79% with uterine contractions, 69% intact membranes, 10% previous CS		4cm/4h (WHO Modified partograph)	Resuscitation defined as artificail ventilation, heart massage and drug use. Not timing or indications for resuscitation provided.<br>No neonatal deaths reported in the study population<br>233 women meeting the inclusion criteria out of 499 births during study period

India (Shinde, 2012)	Live fetus, singleton, at term (37-40 weeks), cephalic presentation	_	_	_	_	4cm/4h (WHO Modified partograph)	50 nulliparous women and 50 parous women randomilly selected durign a two years period. All women were given water enema. One neonatal death due to severe brith asphyxia.
India (Sanyal, 2014)	Live fetus, singleton, at term, cephalic presentation<br>85% between 38-40 weeks	16%	17%	18%	_	4cm/4h (WHO Modified partograph)	Reported on maternal outcomes not included in this review (PPH, fever, puerperal infection, blood transfusion, wound complication), and admission to NICU<br>No fresh stillbirths or maternal deaths observed<br>Early neonatal mortality was due to neonatal sepsis and meconium aspiration
India (Rani, 2016)	Singleton pregnancy, at term (37-42), cephalic presentation<br>49% 2501-3000 grams	15%	_	_	_	Not specified, probably at 3cm/4h	_
India (Shah, 2016)	Live fetus, singleton, at term, cephalic presentation	17% primigravidae, 3% multigravidae			_	4cm/4h (WHO Modified partograph)	Neonatal asphyxia defined as Apgar score of <7 at 0,1,5 min<br>The labour protocol carried out was as follows: Per vaginal examinations were carried out on admission, and at 4 hourly intervals or more frequently if needed. Maternal and fetal parameters were plotted on the modified WHO partograph in active labour. When the progress appeared normal on partograph no intervention was made. When the plot crossed the alert line the patient was immediately reassessed. Oxytocin augmentation was considered when the alert line was crossed or at 6 hours after admission if the uterine contractions were inadequate (defined as contractions of <4 per 10 min lasting for <40 sec). When the plot crossed action line immediate intervention in form of caesarean section was done
Nigeria and Uganda (Souza, 2018)*	Live fetus, singleton, at near-term ot term (34 weeks or more)<br>98.6 cephalic presentation	13%	_	35%	90% spontaneous labour, 12% pre-labour complications during pregnancy, 2% pharmacological analgesia, 5.4% previous CS	4cm/4h (WHO Modified partograph)	Women who were unwilling to participate or incapable of giving consent due to obstetric emergencies were excluded<br>Analysis included women with at least two assessments of cervical dilatation
