# Supplementary material for: Diagnostic accuracy of the partograph alert and action lines to predict adverse birth outcomes: a systematic review
Source: BJOG. 2019 Aug 18;126(13):1524–33. doi: 10.1111/1471-0528.15884 (PMC6899985; doi:10.1111/1471-0528.15884)
Supplement: Supplementary file 3 — Table S2. Suggested interpretation of diagnostic accuracy statistics. [file BJO-126-1524-s003.pdf]

Table S2. Suggested interpretation of diagnostic accuracy statistics

Interpretation	Positive Likelihood Ratio	Negative Likelihood Ratio	Diagnostic Odds Ratio	J statistic [Youden's Index]
Non-discrimination	1.00	1.00	1.00	0.00
Poor	1.01-1.50	0.99-0.67	1.01-2.25	0.01-0.20
Intermediate	1.51-9.99	0.66-0.11	2.26-99.99	0.21-0.79
Excellent	≥10.00	≤0.10	≥100.00	≥0.80
