# Supplementary material for: Diagnostic accuracy of the partograph alert and action lines to predict adverse birth outcomes: a systematic review
Source: BJOG. 2019 Aug 18;126(13):1524–33. doi: 10.1111/1471-0528.15884 (PMC6899985; doi:10.1111/1471-0528.15884)
Supplement: Supplementary file 4 — Table S3. Diagnostic test accuracy of the alert line for adverse neonatal outcomes. [file BJO-126-1524-s004.pdf]

Table S3. Diagnostic test accuracy of the alert line for adverse neonatal outcomes

Country (Year) [reference]	Alert line status	Adverse Neonatal Outcome*		Percentage of alert line crossing	Prevalence of adverse neonatal outcome	Sensitivity (95% CI)	Specificity (95% CI)	Positive Likelihood Ratio (95% CI)	Negative likelihood ratio (95% CI)	Diagnostic Odds Ratio (95% CI)	J statistic (95% CI)
		Present	Absent								
Apgar Score at 1 min <7											
Indonesia, Malaysia and Thailand (1994) [WHO]	Crossed	209	1323	23.8%	6.0%	32.0%	26.7%	2.5	0.6	4.3	32.6%
	Not crossed	175	4735			(49.4-59.3)	(77.1-79.2)	(2.3-2.8)	(0.5-0.7)	(3.5-5.3)	(27.5-37.7)
Mali (2009) [Diarra**]	Crossed	107	441	36.0%	9.6%	73.3%	68.0%	2.3	0.4	5.8	41.2%
	Not crossed	39	935			(65.6-79.8)	(65.4-70.4)	(2.0-2.6)	(0.3-0.5)	(4.0-8.5)	(33.6-48.8)
India(2014) [Sanyal]	Crossed	63	33	19.2%	26.0%	48.5%	91.1%	5.4	0.6	9.6	39.5%
	Not crossed	67	337			(40.0-57.0)	(87.7-93.6)	(3.8-7.9)	(0.5-0.7)	(5.9-15.8)	(30.5-48.6)
Iran (2006) [Bolbol-Haghighi]	Crossed	10	30	29.4%	9.6%	76.9%	75.6%	3.2	0.3	10.3	52.5%
	Not crossed	3	93			(49.7-91.8)	(67.3-82.4)	(2.1-4.9)	(0.1-0.8)	(2.7- 40.0)	(28.4-76.7)
Overall***	Crossed	180	504	31.7%	13.4%	34.8%	82.8%	2.0	0.8	2.6	17.6%
	Not crossed	109	1365			(32.5-37.1)	(81.7-83.9)	(1.9-2.2)	(0.7-0.8)	(2.7-2.9)	(15-20.1)
Apgar Score at 5min <7											
South Africa (2006) [Van Bogaert]	Crossed	28	433	75.9%	7.6%	60.9%	22.8%	0.79	1.72	0.46	-16.3%
	Not crossed	18	128			(46.5-73.6)	(19.5-26.5)	(0.6-1.0)	(1.2-2.5)	(0.3-0.9)	(-30.8-(-1.8))
Ecuador (2008) [López]	Crossed	3	289	58.4%	0.6%	100.0%	41.9%	1.72	NA	NA	
	Not crossed	0	208			(43.9-100)	(37.6-46.2)	(1.6-1.9)			
Brazil (2009) [Rocha]	Crossed	2	98	42.9%	1.3%	66.7%	57.4%	1.56	0.58	2.69	24.1%
	Not crossed	1	132			(20.8-93.6)	(50.9-63.6)	(0.7-3.5)	(0.1-2.9)	(0.2-30.1)	(-29.6-77.8)
India(2014) [Sanyal]	Crossed	43	53	19.2%	17.2%	50.0%	87.2%	3.91	0.57	6.81	37.20%
	Not crossed	43	361			(39.7-60.3)	(83.6-90.0)	(2.8-5.4)	(0.5-0.7)	(4.1-11.4)	(26.2-48.2%)
India (2016) [Rani]	Crossed	4	109	56.5%	2.0%	100.0%	44.4%	1.80	NA	NA	44.4%
	Not crossed	0	87			(51.0- 100)	(37.6-51.4)	(1.6-2.0)			(37.4-51.3)
Nigeria and Uganda (2018) [Souza]	Crossed	143	3990	49.0%	2.8%	60.9%	51.4%	1.25	0.76	1.64	12.2%
	Not crossed	92	4215			(54.5-66.9)	(50.3-52.5)	(1.1-1.4)	(0.7-0.9)	(1.3-2.1)	(5.9-18.6)
Overall	Crossed	223	4972	49.6%	3.6%	59.2%	50.8%	1.20	0.80	1.49	9.9%
	Not crossed	154	5131			(54.1-64.0)	(49.8-51.8)	(1.1- 1.3)	(0.7- 0.9)	(1.2- 1.8)	(4.9-15.0)
**Neonatal resuscitation********											
Senegal (1992) [Dujardin]	Crossed	14	67	8.4%	5.4%	26.9%	92.6%	3.7	0.8	4.6	0.2%
	Not crossed	38	842			(16.8-40.3)	(90.8-94.2)	(2.2-6.0)	(0.7-0.9)	(2.4-9.0)	(7.4-31-7)

South Africa (2006) [Van Bogaert]	Crossed	82	379	75.9%	21.9%	61.7%	20.0%	0.8	1.9	0.4	-0.2%
	Not crossed	51	95			(53.2-69.5)	(16.7-23.9)	(0.7-0.9)	(1.5-2.5)	(0.3-0.6)	(-27.3-(-9.3))
Brazil (2009)[Rocha]	Crossed	4	96	42.9%	2.6%	66.7%	57.7%	1.6	0.6	2.7	0.2%
	Not crossed	2	131			(30.0-90.3)	(51.2-64.0)	(0.9-2.8)	(0.2-1.8)	(0.5-15.2)	(-13.9-62.6)
Nigeria and Uganda (2018)[Souza]	Crossed	34	4099	49.0%	0.7%	54.0%	51.1%	1.1	0.9	1.2	0.1%
	Not crossed	29	4278			(41.8-65.7)	(50.0-52.1)	(0.9-1.4)	(0.7-1.2)	(0.7-2.0)	(-7.3-17.4)
Birth asphyxia											
Nigeria (2008) [Orji 2008]	Crossed	22	186	45.4%	10.3%	46.8%	54.7%	1.0	1.0	1.1	0
	Not crossed	25	225			(33.3-60.8)	(49.9-59.5)	(0.8-1.4)	(0.7-1.3)	(0.6-2.0)	(-13.5-16.6)
India (2016)[Rani]	Crossed	7	106	56.5%	4.5%	77.8%	44.5%	1.4	0.5	2.8	0.2%
	Not crossed	2	85			(45.3-93.7)	(37.6-51.6)	(0.97-2.0)	(0.2-1.7)	(0.6-13.9)	(-5.8-50.3)
India (2016)[Shah]	Crossed	7	79	32.8%	5.7%	46.7%	68.0%	1.5	0.8	1.9	0.1%
	Not crossed	8	168			(24.8-69.9)	(62.0-73.5)	(0.8-2.6)	(0.5-1.3)	(0.7-5.3)	(-11.2-40.6)
Overall	Crossed	29	292	48.8%	8.5%	51.8%	51.5%	1.1	0.9	1.1	0
	Not crossed	27	310			(39.0-64.3)	(47.5-55.5)	(0.8-1.4)	(0.7-1.2)	(0.7-2.0)	(-10.4-17.0)
Early neonatal mortality											
South Africa (2006)***** [Van Bogaert]	Crossed	8	453	75.9%	3.0%	44.4%	23.1%	0.6	2.4	0.2	-0.3%
	Not crossed	10	136			(24.6-66.3)	(19.9-26.7)	(0.3-0.97)	(1.6-3.7)	(0.1-0.6)	(-55.7-(-9.3))

NA: not applicable
** Stillbirths and unknown outcomes excluded from the denominator as follows; Dujardin 1992: outcome unknown for 33 neonates and 28 stillbirths excluded; WHO 1994: 3 stillbirths excluded; Van Bogaert 2006: 3 stillbirths excluded Orji 2008: 5 stillbirths excluded; Souza 2018: 49 stillbirths excluded)*
***According to data published in full thesis at: http://www.keneya.net/fmpos/theses/2007/med/pdf/07M235.pdf.,and confirmed by the authors.*

****WHO 1994 excluded as reported Apgar Score at 1 min <8*

*****Resuscitation definitions vary between studies and results were not pooled. VanBogaert: Resuscitation determined by an Apgar score at 1 min of less than 7; Rocha: artificial ventilation, heart massage, drug use, timing not specified*
****** Deaths resulted from low Apgar scores and unsuccessful resuscitation*
