# Supplementary material for: Diagnostic accuracy of the partograph alert and action lines to predict adverse birth outcomes: a systematic review
Source: BJOG. 2019 Aug 18;126(13):1524–33. doi: 10.1111/1471-0528.15884 (PMC6899985; doi:10.1111/1471-0528.15884)
Supplement: Supplementary file 5 — Table S4. Diagnostic test accuracy of the action line for adverse fetal outcomes. [file BJO-126-1524-s005.pdf]

Table S4. Diagnostic test accuracy of the action line for adverse fetal outcomes

Country (Year) reference	Action line status	Adverse Fetal Outcome		Percentage of action line crossing	Prevalence of adverse fetal outcomes	Sensitivity (95% CI)	Specificity (95% CI)	Positive likelihood ratio (95% CI)	Negative likelihood ratio (95% CI)	Diagnostic Odds Ratio (95% CI)	J statistic (95% CI)
		Present	Absent								
Senegal (1992) Dujardin	Crossed*	3	28	3.1%	1.4%	21.4%	97.2%	7.55	0.81	9.33	18.6%
	Not crossed**	11	958			(7.6-47.6)	(95.9-98.0)	(2.6-21.9)	(0.6-1.1)	(2.5-35.3)	(-2.9-40.1)
Indonesia, Malaysia and Thailand (1994) WHO	Crossed	0	521	8.1%	0.0%	0.0%	91.9%	NA	1.09	NA	-8.1%
	Not crossed	3	5921			(0-56.15)	(91.2-93.0)		(1.1-1.1)		(-0.1-(-0.07))
Nigeria (2008) Orji	Crossed	0	102	22.0%	1.1%	0	0.8	NA	1.3	NA	-22.3%
	Not crossed	5	356			(0-43.5)	(73.7-81.3)		(1.2-1.4)		(-0.3-(-0.2))
Nigeria and Uganda (2018) Souza	Crossed	9	1259	14.9%	0.6%	18.4%	85.1%	1.23	0.96	1.28	3.5%
	Not crossed	40	7181			(10.0-31.4)	(84.3-85.8)	(0.7-2.2)	(0.8-1.1)	(0.6- 2.7)	(-0.1-0.1)

NA not applicable; *outcome unknown for 4 women and 0 macerated stillbirths excluded from the denominator; **outcome unknown for 4 women and 14 macerated stillbirths excluded from the denominator.
