# Supplementary material for: Diagnostic accuracy of the partograph alert and action lines to predict adverse birth outcomes: a systematic review
Source: BJOG. 2019 Aug 18;126(13):1524–33. doi: 10.1111/1471-0528.15884 (PMC6899985; doi:10.1111/1471-0528.15884)
Supplement: Supplementary file 6 — Table S5. Diagnostic test accuracy of the action line for adverse neonatal outcomes. [file BJO-126-1524-s006.pdf]

Table S5. Diagnostic test accuracy of the action line for adverse neonatal outcomes

Country (Year) reference	Action line status	Adverse Neonatal Outcome		Percentage of action line crossing	Prevalence of adverse neonatal outcome	Sensitivity (95% CI)	Specificity (95% CI)	Positive likelihood ratio (95% CI)	Negative likelihood ratio (95% CI)	Diagnostic Odds Ratio (95% CI)	J statistic (95% CI)
		Present	Absent								
Apgar Score at 1 min <7											
Indonesia, Malaysia and Thailand (1994)	Crossed	98	423	8.1%	6.0%	25.5%	93.0%	3.65	0.80	4.56	18.5%
WHO [Apgar Score at 1 min <8]	Not crossed*	286	5635			(21.4-30.1)	(92.4-93.6)	(3.0-4.4)	(0.8-0.9)	(3.6-5.9)	(14.1-22.9)
Mali (2009) Diarra**	Crossed	62	10	4.7%	9.6%	42.5%	99.3%	58.43	0.58	100.82	41.7%
	Not crossed	84	1366			(34.7-50.6)	(98.7-99.6)	(30.6-111.5)	(0.5-0.7)	(49.9-203.7)	(33.7-49.8)
Apgar Score at 5min <7											
Ecuador (2008) López	Crossed	0	64	12.8%	0.0%	NA	NA	NA	NA	NA	NA
	Not crossed	0	436								
Brazil (2009) Rocha	Crossed	0	21	9.0%	1.3%	0.0%	90.9%	NA	1.10	NA	-9.1%
	Not crossed	3	209			(0-56.2)	(86.5-94.0)		(1.1-1.2)		(-12.9-(-5.4))
India (2016) Rani	Crossed	0	32	16.0%	2.0%	0.0%	83.7%	NA	1.20	NA	-16.3%
	Not crossed	4	164			(0-49.0)	(77.9-88.2)		(1.2-1.3)		(-21.5-(-11.2))
Nigeria and Uganda (2018) Souza	Crossed	51	1217	14.9%	2.8%	21.7%	85.3%	1.47	0.92	1.60	7.0%
	Not crossed	184	7037			(16.9-27.4)	(84.5-86.0)	(1.2-1.9)	(0.9-0.98)	(1.17-2.20)	(1.6-12.3)
**Neonatal resuscitation********											
Senegal (1992) Dujardin	Crossed	4	19	2.4%	5.4%	7.7%	97.9%	3.68	0.94	3.90	5.6%
[Neonatal resuscitation at birth]	Not crossed	48	890			(3.0-18.2)	(96.898.7)	(1.3-10.4)	(0.87-1.02)	(1.3-11.9)	(-1.7-12.9)
Brazil (2009) Rocha	Crossed	0	21	9.0%	2.6%	0.0%	90.7%	NA	1.10	NA	-9.3%
[Neonatal resuscitation]	Not crossed	6	206			(0-39.0)	(86.3-93.9)		(1.06-1.15)		(-13.0-(-5.5))
Nigeria and Uganda (2018) Souza	Crossed	14	1254	14.9%	0.7%	22.2%	85.1%	1.49	0.91	1.63	7.3%
[Neonatal resuscitation during hospital stay]	Not crossed	49	7172			(13.7-33.9)	(84.3-85.9)	(0.9-2.4)	(0.8-1.0)	(0.9-3.0)	(-3.0-17.6)
Birth asphyxia											
Nigeria (2008) Orji	Crossed	13	89	22.0%	10.2%	27.7%	78.6%	1.29	0.92	1.40	6.3%
	Not crossed	34	327			(16.9-41.8)	(74.4-82.3)	(0.79-2.13)	(0.77-1.11)	(0.71-2.8)	(-7.1-19.6)
India (2016) Shah	Crossed	1	8	3.5%	5.0%	7.7%	96.7%	2.37	0.95	2.48	4.4%
	Not crossed	12	238			(13.7-33.3)	(93.7-98.3)	(0.3-17.5)	(0.8-1.1)	(0.3-21.5)	(-10.2-19.1)
India (2016) Rani	Crossed	0	32	13.1%	3.7%	0.0%	86.4%	NA	1.16	NA	-13.6%
	Not crossed	9	203			(0-29.9)	(81.4-90.2)		(1.1-1.2)		(-18.0-(-9.2))

NA: not applicable; * Stillbirths and unknown outcomes excluded from the denominator as follows: Dujardin 1992: outcome unknown for 33 neonates and 28 stillbirths excluded; WHO 1994: 3 stillbirths;**According to data published in full thesis at: http://www.keneya.net/fmpos/theses/2007/med/pdf/07M235.pdf and confirmed by the authors; ****Resuscitation definitions vary between studies and results were not pooled. VanBogaert: Resuscitation determined by an Apgar score at 1 min of less than 7; Rocha: artificial ventilation, heart massage, drug use, timing not specified
