# Supplementary material for: Diagnostic accuracy of the partograph alert and action lines to predict adverse birth outcomes: a systematic review
Source: BJOG. 2019 Aug 18;126(13):1524–33. doi: 10.1111/1471-0528.15884 (PMC6899985; doi:10.1111/1471-0528.15884)
Supplement: Supplementary file 7 — Table S6. Diagnostic test accuracy of the alert line for composite adverse birth outcomes. [file BJO-126-1524-s007.pdf]

Table S6. Diagnostic test accuracy of the alert line for adverse composite birth outcomes

Country (Year) reference [Outcome]	Alert line status	Adverse Composite Birth Outcome (ABO)		Percentage of alert line crossing	Prevalence of ABO	Sensitivity (95% CI)	Specificity (95% CI)	Positive likelihood ratio (95% CI)	Negative likelihood ratio (95% CI)	Diagnostic Odds Ratio (95% CI)	J statistic (95% CI)
		Present	Absent								
Senegal (1992)[Dujardin]	Crossed	19	62	8.4%	6.8%	28.8%	93.1%	4.18	0.76	5.47	21.9%
[fresh stillbirths or neonatal resuscitation at birth]	Not crossed	47	839			(19.3-40.6)	(91.3 -94.6)	(2.7 -6.6)	(0.7-0.9)	(3.0-9.9)	(10.9-33.0)
Indonesia, Malaysia and Thailand (1994)[WHO]	Crossed	209	1323	16.6%	3.8%	54.0%	78.2%	2.47	0.59	4.20	32.2%
[fresh stillbirths or Apgar score<8 at one minute among women admitted in active phase]	Not crossed	178	4735			(49.0-58.9)	(77.1-79.2)	(2.2-2.7)	(0.5-0.7)	(3.4-5.2)	(27.1-37.2)
South Africa (2006)[Van Bogaert]	Crossed	30	433	75.9%	8.0%	61.2%	22.8%	0.79	1.7	0.47	-16.0%
[fresh stillbirths or Apgar score<7 at five minutes]	Not crossed	19	128			(47.3-73.6)	(19.5-26.5)	(63.2-99.6)	(1.2-2.5)	(0.3-0.9)	(-30.0 - (-1.9))
Nigeria (2008)[Orji]	Crossed	27	186	46.0%	11.2%	51.9%	54.7%	1.15	0.88	1.31	6.7%
[fresh stillbirth or birth asphyxia]	Not crossed	25	225			(38.7-64.9)	(49.9-.59.5)	(0.9-1.5)	(0.7-1.2)	(0.7-2.3)	(-7.7-21.1)
Nigeria and Uganda (2018)[Souza]	Crossed	152	4011	49.0%	3.0%	59.8%	51.3%	1.23	0.78	1.57	11.1%
[fresh stillbirth or Apgar Score at 5 min <7 or neonatal resuscitation during hospital stay]	Not crossed	102	4224			(53.7-65.7)	(50.2-52.4)	(1.1-1.4)	(0.7-0.9)	(1.2-2.0)	(5.0-17.3)
Nigeria and Uganda (2018)[Souza]	Crossed	110	4053	49.0%	2.3%	56.7%	51.1%	1.16	0.85	1.37	7.8%
*[severe adverse birth outcomes]**	Not crossed	84	4242			(49.7-63.5)	(50.1-52.2)	(1.0-1.3)	(0.7-100)	(1.0-1.8)	(0.8-14.9)

*ABO: Adverse birth outcome; NA: not applicable; * Severe adverse birth outcomes were defined as the occurrence of any of the following: stillbirths, intra-hospital early neonatal deaths, neonatal use of anticonvulsants, neonatal cardio-pulmonary resuscitation, Apgar score < 6 at 5 minutes, uterine rupture and maternal death or organ dysfunction with dystocia.*
