# Supplementary material for: Diagnostic accuracy of the partograph alert and action lines to predict adverse birth outcomes: a systematic review
Source: BJOG. 2019 Aug 18;126(13):1524–33. doi: 10.1111/1471-0528.15884 (PMC6899985; doi:10.1111/1471-0528.15884)
Supplement: Supplementary file 8 — Appendix S1. Search strategy. [file BJO-126-1524-s008.pdf]

Appendix S1. Search strategies, Date 27 April 2017 (1) and 12 February 2019 (2)

#	Database : PubMed<br><br>Database Provider : http://www.pubmed.gov<br><br>Date limits : Language limits :<br>Other notes on the search :	Results
1	Partograh* OR Partogram*	347
2		20

#	Database : EMBASE<br><br>Database Provider : http://www.embase.com<br>Date limits : none<br>Language limits :<br>Other notes on the search :	Results
1	Partograph* OR partogram*	473
		43

#	Database : Global Index Medicus (GIM)<br><br>Database Provider : http://www.globalhealthlibrary.net/<br><br>Date limits : none<br>Language limits : none<br>Other notes on the search : Filter activated to limit to Regional Databases (AIM, LILACS, IMEMR, IMSEAR & WPRIM) Breakdown<br>• LILACS (Americas) (60)<br>• IMEMR (Eastern Mediterranean) (18)<br>• IMSEAR (South-EastAsia) (12)<br>• AIM (Africa) (9)<br>• WPRIM (Western Pacific) (8)	Results
1	Partograph* OR partogram*	107
2		8
#	Database : Web of Science<br><br>Database Provider : http:// apps.webofknowledge.com/<br><br>Date limits : none<br>Language limits : none	Results

	Other notes on the search :	
1	Partograph* OR partogram*	421

#	Database : Popline<br><br>Database Provider : http://www.popline.org/<br><br>Date limits : none<br>Language limits : none<br>Other notes on the search :	Results
1	Partograph* OR partogram*	148
2		17

#	Database : LILACS<br><br>Database Provider : http://lilacs.bvsalud.org/<br>Date limits : none<br>Language limits : none<br>Other notes on the search :)	Results
1	Partograph* OR partogram* OR partograf*	69
2		8

#	Database : Central<br><br>Database Provider : http://www.cochranelibrary.com/<br>Date limits : none<br>Language limits : none<br>Other notes on the search :)	Results
1	Partograph* OR partogram*	38
2		13

#	Database : WPRIM<br><br>Database Provider : http://www.wprim.org/<br>Date limits : none<br>Language limits : none	Results

	Other notes on the search :)	
1	Partograph* OR partogram*	8

#	Database : IMEMR<br><br>Database Provider :<br>http://applications.emro.who.int/library/Databases/wxis.exe/Library/Databases/iah/?IsisScript=iah/iah.xis&lang=I&base=imemr<br>Date limits : none<br>Language limits : none<br>Other notes on the search :)	Results
1	Partograph* OR partogram*	7

#	Database : Ebsco Multi-Database Search<br><br>Database Provider : http://search.ebsco.com<br>Date limits : none<br>Language limits : Medline Citations removed from CINAHL Plus with Full Text and CINAHL Complete<br><br>Other notes on the search :<br>Academic Search Premier 134<br>Academic Search Complete 134<br>Health Source: Nursing/Academic Edition 41<br>CINAHL Plus with Full Text 41<br>CINAHL Complete 41<br>Women's Studies International 25<br>Consumer Health Complete – EBSCOhost 20<br>MasterFILE Premier 12<br>Gender Studies Database 11<br>SocINDEX with Full Text 8<br>Vocational and Career Collection 7<br>Public Affairs Index 6<br>Business Source Premier 5<br>Business Source Complete 5<br>PsycINFO 4<br>Psychology and Behavioral Sciences Collection 4<br>Environment Complete 4<br>Health Source - Consumer Edition 3<br>SPORTDiscus with Full Text 3<br>Food Science Source 3<br>ERIC 2<br>Middle Eastern & Central Asian Studies 2<br>Alt HealthWatch 1<br>Regional Business News 1<br>MedicLatina 1	Results

	Agricola 1<br>Legal Collection 1<br>Library, Information Science & Technology Abstracts with Full Text<br>1<br>Education Research Complete 1<br>Risk Management Reference Center 1<br>Caribbean Search 1	
1	Partograph* OR partogram*	189
2		61

#	Database : CAJD<br><br>Database Provider : http://oversea.cnki.net/kns55/brief/result.aspx?dbPrefix=CJFD<br>Date limits : none<br>Language limits : none<br>Other notes on the search : Search limited due to variance in translation of “partograph or partogramme” into Chinese.	Results
1	TI=partogram OR TI='产程图' OR TI=Partograms OR TI=Partographs OR TI=partograph OR KY=partogram OR KY=Partograms OR KY=Partographs OR KY=partograph	91
2		1

#	Database : eLibrary.ru<br><br>Database Provider : http://elibrary.ru/defaultx.asp<br>Date limits : none<br>Language limits : none<br>Other notes on the search : Not able to import citations into EndNote. Viewed only online	Results
1	Партограмма OR ПАРТОГРАММЫ	73

#	Database : African Index Medicus<br><br>Database Provider : http://indexmedicus.afro.who.int/<br>Date limits : none<br>Language limits : none<br>Other notes on the search : Not able to import citations into EndNote. Viewed only online	Results
1	Partograph* partogram*	11
2		2

#	Database : IMSEAR<br><br>Database Provider : http://imsear.li.mahidol.ac.th/<br>Date limits : none<br>Language limits : none<br>Other notes on the search :	Results
1	TITLE : Partograph* partogram*	5
	SUBJECT: Partograph* partogram*	2
2		1

#	Database : SCOPUS<br><br>Database Provider : https://www.scopus.com/home.uri<br>Date limits : none<br>Language limits : none<br>Other notes on the search :	Results
2	Partograph* partogram*	68
